# Altered brain network dynamics and functional connectivity in subjective cognitive decline: an edge-centric network study

**DOI:** 10.3389/fnagi.2025.1596537

**Published:** 2026-01-09

**Authors:** Xiaofan Wei, Baiwan Zhou, Juanling Li, Ruohong Xu, Wei Zhang

**Affiliations:** 1 Department of Radiology, The Second Affiliated Hospital of Chongqing Medical University, Chongqing, China; 2 Department of Radiology, Xichang People’s Hospital, Liangshan, Sichuan, China

**Keywords:** subjective cognitive decline, resting-state functional magnetic resonance imaging, edge-centric network, high- and low-amplitude frame network, dynamic functional connectivity

## Abstract

**Purpose:**

To explore neurodynamic bases underlying subjective cognitive decline (SCD) based on edge-centric functional network.

**Methods:**

211 SCD patients and 210 healthy controls (HC) were recruited from the Alzheimer’s Disease Neuroimaging Initiative. Edge time series (ETS) were obtained based on resting-state functional magnetic resonance data. The top 10% co-fluctuation signals of all time points in ETS were extracted to construct the high-amplitude frame networks, and the co-fluctuation signals from the remaining time points were used to construct the low-amplitude frame networks. In both network states, the graph theory and network-based statistics (NBS) analyses were used to compare SCD and HC. The correlation of the imaging indicators with cognitive scores and apolipoprotein E (APOE) ε4 genes was performed by Spearman correlation analysis.

**Results:**

SCD exhibited lower peak amplitude and longer trough-to-trough duration (TTD) compared to HC. In both network states, the normalized clustering coefficient, normalized characteristic path length, small-worldness, and global efficiency of SCD were significantly reduced, and the altered nodal centralities of SCD predominantly exhibited a decreasing trend. However, the high-amplitude frame network identified more altered brain regions compared to the low-amplitude frame network. Furthermore, a SCD-related subnetwork was found in the high-amplitude frame network, which was composed of 11 brain regions and 13 edges. TTD was positively related to the number of APOE ε4 genes; the normalized characteristic path length, the betweenness centrality of right postcentral gyrus, and the connection between bilateral angular gyrus were correlated with cognitive scores.

**Conclusion:**

Our findings demonstrate that the edge-centric network framework reveals details of brain network alterations in SCD through different perspectives, and these alterations hold potential as novel biomarkers for SCD.

## 1 Introduction

Subjective cognitive decline (SCD) is defined as an individual’s self-perception of cognitive decline with normal performance on standardized cognitive tests (Jessen et al., 2020; Lin et al., 2019). The prevalence of SCD is as high as 44.5% in elderly people over 60 years old, and the risk of progression to dementia is 2–3 times higher than that of healthy subjects (HC) (Kang et al., 2024; Liew, 2020; Pike et al., 2022). Multiple studies on biomarkers related to Alzheimer’s disease (AD) have shown that the carrier rate of apolipoprotein E (APOE) ε4 genes in SCD patients is significantly higher than that in HC, and that amyloid-β (Aβ) and tau protein have already begun to aggregate during the SCD stage, with the extent of their deposition positively correlating with the severity of cognitive impairment in patients (Bolton et al., 2024; Pérez-Cordón et al., 2020; Wang et al., 2020). This evidence suggests that SCD, as a prodromal manifestation of AD-related cognitive impairment, may be an effective entry point to delay the progression of AD. However, due to the mild manifestations of SCD and its definition based on subjective feelings, its clinical diagnosis is not yet unified (Jessen et al., 2014). Therefore, exploring the objective neural mechanism behind self-perceived cognitive decline is essential for refining the diagnostic criteria and constructing early biomarkers for SCD.

Human behavior and cognition originate from complex interactions in the brain, which are driven by the connectivity of local and distant regions in the brain network (Behrens and Sporns, 2012; Thiebaut de Schotten and Forkel, 2022). At present, resting-state functional magnetic resonance imaging (rs-fMRI) is an important method for noninvasive assessment of functional connectivity (FC) in the brain (Blamire, 2018; Risacher and Saykin, 2019). Early rs-fMRI studies have revealed preclinical network abnormalities in SCD, mainly manifested as a decrease in neural activity or disrupted FC in vulnerable brain regions such as the hippocampus (Serra et al., 2023; Wang et al., 2022). In recent years, growing evidence indicates that FC exhibits significant fluctuations over time (Gao et al., 2020). These dynamic FC changes are thought to embed features associated with behavior and cognition. Existing studies have shown that the dynamic FC network has been restructured during the stage of SCD, and the altered dynamic FC properties are significantly correlated with cognitive performance (Chen et al., 2021). As the field advances, the focus of dynamic FC research is evolving: from exploring its role in adaptive cognition and behavior, toward detecting these dynamics in individuals during complex cognitive tasks — a process demanding sufficiently high temporal and spatial resolution (Cohen, 2018). In most previous studies, dynamic FC was assessed by sliding window method. However, due to the diversity of window parameter selection and the blurring effect caused by the windowing procedure, this method cannot accurately localize changes at specific moments in FC. These limitations hinder our understanding of dynamic FC.

In 2020, Faskowitz et al. (2020) developed a novel edge-centric functional connectivity (eFC) network framework, also known as co-fluctuation or edge time series (ETS). On the one hand, the eFC method can accurately decompose each edge (connection) into moment-to-moment co-fluctuations across time, intuitively linking patterns of edge cofluctuations to fine-scale dynamics of FC (Sporns et al., 2021). This advantage of this method makes it possible to analyze alterations in brain dynamics at an individual time point (Sporns et al., 2021). Therefore, functional properties assessed at single fMRI time-point resolution may more accurately reflect the relationship between FC dynamics and neurocognitive outcomes, compared to conventional “full FC” or FC within specific time windows. On the other hand, unlike traditional node-centric functional connectivity (nFC) analysis, the measurement of correlation in eFC analysis can be understood as a “talk” between pairs of edges rather than pairs of brain regions (Sun et al., 2023). Consequently, eFC may provide important complementary information for disease diagnosis. Recently, several studies based on the eFC method fully revealed the effectiveness of co-fluctuations information in identifying patients with autism spectrum disorders (ASD), and the ASD classification model constructed by the eFC significantly improved the classification performance (Sun et al., 2023; Zamani Esfahlani et al., 2022). Similarly, Jiang et al. (2022) found that edge-centric networks better differentiated opioid-exposed infants from controls, and changes in co-fluctuations between edges may be related to the neural basis of opioid-exposed infants. In addition, a longitudinal dataset acquired multiple measures via eFC, where the normalized entropy was related to the severity of stroke and increased over the course of patient recovery (Idesis et al., 2022). These results confirm that the eFC method can be used as an effective tool for the classification and diagnosis of a variety of diseases, which is regarded as an important supplement to traditional methods. While the role of moment-to-moment co-fluctuations in characterizing functional connectivity patterns in SCD remains poorly understood, we hypothesize that applying eFC to SCD will reveal details of SCD-associated alterations in functional brain dynamics from different perspectives.

Based on the above, the aim of this study is to use functional data to obtain ETS, separate time points according to co-fluctuation amplitude, and construct high- and low-amplitude co-fluctuation networks to distinguish the differences in brain dynamics and network metrics between SCD and HC, and to further explore the correlations between network metrics, cognitive scores, and APOE ε4 genes.

## 2 Materials and methods

### 2.1 Participants

A total of 421 subjects were selected from the Alzheimer’s Disease Neuroimaging Initiative (ADNI) database,^1^ including 211 SCD patients and 210 HC. The ADNI-2 procedures manual describes specific inclusion and diagnostic criteria; for more details, please refer to http://www.adni-info.org/Scientists/ADNIStudyProcedures.html. In brief, SCD patients exhibit subjective memory concerns, the score of the first 12 items of the Cognitive Change Index > 16, the Mini-Mental State Examination (MMSE) scores between 24 and 30, and Clinical Dementia Rating Scale (CDR) = 0; HC do not report any subjective memory complaints and demonstrate normal cognitive performance.

### 2.2 Clinical data and neuropsychological tests

We collected the clinical and laboratory data of the subjects from the ADNI website, including age, gender, education level, and the number of APOE ε4 genes. The Montreal Cognitive Assessment (MOCA) and the MMSE were used to assess the baseline cognitive function of all subjects.

### 2.3 rs-fMRI data acquisition and preprocessing

Image acquisition for 421 subjects was performed using a variety of scanners. The scanning parameters varied slightly across different scanner models; for further details, please refer to the Supplementary materials. Additional information regarding image acquisition protocols can be accessed on the ADNI website^2^.

All functional data were processed using MATLAB R2017a and GRETNA (V2.0). The preprocessing steps included the following: (1) conversion of DICOM data into NIFTI format; (2) the first 10 volumes of NIFTI format were discarded to achieve a steady state; (3) the remaining volumes were corrected for differences in slice acquisition times; (4) the individual images were realigned so that each part of the brain in each volume is in the same position; (5) all functional images were normalized to the standard Montreal Neurological Institute (MNI) space using the echo-planar imaging (EPI) template, resampled to 3 × 3 × 3 mm^3^ voxels, and spatially smoothing with a Gaussian kernel (full width at half-maximum of 4 mm); (6) linear detrend were conducted; (7) to minimize noise contamination, signals from cerebrospinal fluid, white matter, and head motion parameters were regressed out; (8) a band-pass filter (0.01–0.1 Hz) was applied to reduce the effects of low-frequency drift and high-frequency physiological noises.

### 2.4 Edge time series and its measurement

Firstly, the preprocessed functional data was parcellated into 200 regions of interest (ROI) using the Schaefer 200 atlas (Schaefer et al., 2018). The core algorithm of traditional network construction is completed by Pearson correlation coefficient (PCC). Its calculation involves summing the products of the z-scores of two time series, which is then divided by the number of time points minus one (T−1).

The z-score normalization of the each time series was calculated as $z_{i}=\frac{x_{i}-u_{i}}{\sigma_{i}}$, where *x*_*i*_ = [*x*_*i*_(1),…,*x*_*i*_(*T*)] and *x*_*j*_ = [*x*_*j*_(1),…,*x*_*j*_(*T*)] are the BOLD time series of parcels *i* and *j*, and T is the time points. *z*_*i*_(*t*) and *z*_*j*_(*t*) are any 2 time series z-scores. Finally, PCC can be expressed as $r_{i\,j}=\frac{1}{T-1}\,\sum_{t}\left[Z_{i}\,\left(t\right)\cdot Z_{j}\,\left(t\right)\right]$. However, the eFC calculation method removes the step of calculating the mean in the process described above. After obtaining the product of the elements of the z-score time series, the values at each time point are listed separately to obtain an edge time series, so that each edge is regarded as a node in the eFC network. The values of the edge time series reflect the pattern of co-fluctuation of brain regions i and j at each time point. The positive co-fluctuation represents the same direction of activity of brain region i and j, while the negative co-fluctuation reflects the opposite direction of their activity. Finally, we estimated the ETS for all pairs of ROIs to create an edge-by-edge matrix, which are normalized to the interval [−1, 1].

For a single time point, we extract all edge co-fluctuation values to form a curve. Then, the sum square root (RSS) of all co-fluctuation values at this time point was calculated to quantify the global brain co-fluctuation strength at this time point. Next, we calculated the RSS for all ETS at each time point and plotted this value as a time function. Based on the RSS signals, we identified the peaks and troughs, which correspond to specific time points. A trough is identified as a time point where its amplitude is lower than that of its two immediate neighbors. A peak, conversely, corresponds to the time point of the highest amplitude signal between two consecutive troughs. Based on the above, we defined two metrics, peak amplitude and trough-to-trough duration (TTD). Peak amplitude is the highest peak between two troughs, and TTD represents the time interval between two adjacent troughs. Peak amplitude and TTD reflect the degree of brain activity and the flexible state of brain networks, respectively.

### 2.5 Construction and analysis of high-amplitude frame network and low-amplitude frame network

The RSS was calculated for all time points and ranked from high to low. The signals ranked in the top 10% of the time points were retained to evaluate the functional connectivity in the high-amplitude frame state, and the signals from the remaining time points were used to evaluate the functional connectivity in the low-amplitude frame state. The MATLAB-based GRETNA toolbox was employed to analyze the topological properties of high- and low-amplitude frame networks. Referring to previous studies, a wide range of sparsity (S) thresholds (0.05 < S < 0.5, with a step size of 0.05) was applied to each correlation matrix, and network metrics were calculated at each sparsity threshold (Watts and Strogatz, 1998). At the global level, the network indicators examined included the clustering coefficient (Cp), characteristic path length (Lp), normalized characteristic path length (λ), normalized clustering coefficient (γ), small-worldness (σ), global efficiency (Eglob), and local efficiency (Eloc). At the nodal level, nodal efficiency (Ne), degree centrality (Dc), and betweenness centrality (Bc) were measured. Briefly, Cp measures the local interconnectivity extent. Lp is calculated by the average of the shortest path lengths between all possible pairs of nodes in the network. Small-world attributes (γ, λ, and σ) indicate the degree of organization of the small world. Eglob and Eloc indicate the global and nodal efficiency of information transfer in the network, respectively. Dc reflects the importance of the node in the whole brain network, Bc indicates the ability of the node to influence the whole network, and Ne characterizes the efficiency of the ability of node to transmit information in the network.

The network-based statistical (NBS) method was used to investigate the functional connections between brain regions with significant differences in any nodal properties between SCD and HC (*p* < 0.05, T threshold > 2.105). Please refer to the Supplementary materials for more details.

### 2.6 Statistical analysis

The Statistical Package for IBM SPSS Statistics 26 was used for statistical analyses, and the significance threshold was set at *p* < 0.05. Independent-sample *t*-tests and the chi-square test were performed to compare demographic variables between the two groups (SCD and HC). Group comparisons of imaging measures were completed by two-sample *t*-tests (*p* < 0.05). For peak amplitude, TTD, and global topological metrics, ComBat correction was used to reduce scanner and site effects. ComBat Harmonization is a statistical technique based on an empirical Bayesian framework that can be used to analyze data sets obtained with different scanning machines. It is designed to effectively eliminate batch effects in multi-center or multi-batch data sets (Ligero et al., 2021). In our study, we used the Combat package in MATLAB to correct brain function data. For the three nodal metrics, False Discovery Rate (FDR) correction was used for multiple comparisons (*p* < 0.05). Finally, after identifying network indicators with significant between-group differences, Spearman correlation analysis was computed to explore the relationship between these indicators, cognitive scores, and the number of APOE ε4 genes (*p* < 0.05).

## 3 Results

### 3.1 Patients characteristics

Our study included 211 SCD patients and 210 HC. The SCD patients were from 14 sites, and each site had 4, 12, 7, 2, 5, 30, 39, 56, 3, 10, 10, 10, 6, 17 patients. HC came from 13 sites, and each site had 11, 3, 7, 26, 23, 43, 12, 4, 6, 20, 28, 12, 15 persons. There were no significant differences in age, gender, and education level between the two groups (*p* > 0.05). No significant statistical differences were observed in MMSE, MoCA, and the number of APOE ε4 genes. More details are presented in Table 1.

**TABLE 1:** Baseline characteristics of SCD and HC.

Variable	SCD group (*n* = 211)	HC group (*n* = 210)	*P*-value
Age (years)	70.31 ± 7.08	71.36 ± 6.69	0.117
Sex (male/female)	(78/133)	(91/119)	0.183
Education (years)	16.78 ± 2.31	16.60 ± 2.45	0.433
APOE ε4 genes (0,1,2)	(107,61,7)	(137,51,5)	0.132
MMSE	29.12 ± 1.09	29.06 ± 1.14	0.574
MOCA	26.35 ± 2.20	26.30 ± 2.20	0.828

SCD, subjective cognitive decline; HC, healthy controls; APOE, apolipoprotein E; MMSE, the Mini-Mental State Examination; MOCA, the Montreal Cognitive Assessment.

### 3.2 The peak amplitude and trough-to-trough duration in SCD and HC

**FIGURE 1:**
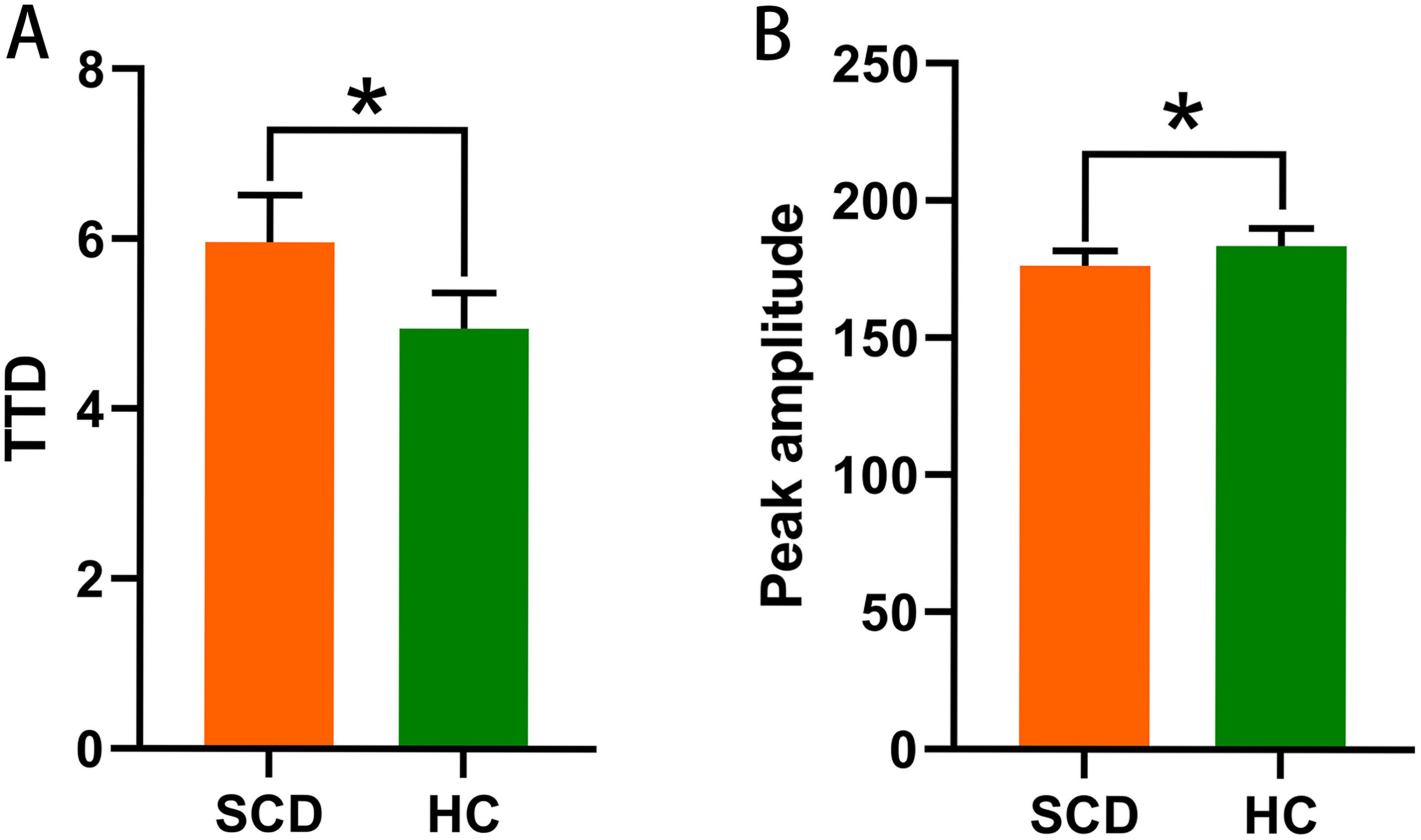
The graphs illustrate differences in brain dynamics characteristics between SCD and HC. Longer TTD **(A)** and lower peak amplitude **(B)** were found in SCD than HC. Asterisks (*) indicate significant between-group differences (*p* < 0.05). SCD, subjective cognitive decline; HC, healthy control; TTD, duration of trough-to-trough.

To determine whether SCD exists functional brain dynamics disruptions, we calculated their mean peak amplitude and TTD, and compared them with HC. As showed in Figure 1 and Table 2, compared with the HC, SCD had lower mean peak amplitude (*p* = 0.020, Cohen’s *d* = −0.227) and longer TTD (*p* < 0.001, Cohen’s *d* = 2.066).

**TABLE 2:** Differences in brain dynamics characteristics and global network metrics between SCD and HC.

Measurements	SCD group (*n* = 211)	HC group (*n* = 210)	df	*P*-value	Cohen’s d (95% CI)
Peak amplitude	179.38 ± 5.37	180.72 ± 6.34	419	0.020*	−0.227 (−0.419, −0.035)
Trough-to-trough duration (TTD)	5.96 ± 0.55	4.95 ± 0.42	419	<0.001*	**2.066 (1.829, 2.302)**
**High-amplitude frames network**					
Clustering coefficient (Cp)	0.28 ± 0.01	0.28 ± 0.01	419	0.303	**−**0.101 (**−**0.292, 0.091)
Normalized clustering coefficient (γ)	0.78 ± 0.14	0.82 ± 0.14	419	0.009*	**−0.260 (−0.451, −0.068)**
Characteristic path length (Lp)	0.89 ± 0.07	0.89 ± 0.06	419	0.721	0.035 (−0.156, 0.226)
Normalized characteristic path length (λ)	0.50 ± 0.02	0.51 ± 0.01	419	0.004*	**−0.200 (−0.392, −0.009)**
Small-worldness (σ)	0.69 ± 0.12	0.72 ± 0.12	419	0.032*	**−0.211 (−0.402, −0.019)**
Global efficiency (Eglob)	0.25 ± 0.01	0.25 ± 0.01	419	0.496	−0.066 (−0.257, 0.125)
Local efficiency (Eloc)	0.35 ± 0.01	0.36 ± 0.01	419	0.015*	**−0.239 (−0.431, −0.047)**
**Low-amplitude frames network**					
Clustering coefficient (Cp)	0.25 ± 0.01	0.25 ± 0.01	419	0.105	−0.158 (−0.350, −0.033)
Normalized clustering coefficient (γ)	0.71 ± 0.07	0.74 ± 0.09	419	0.001*	**−0.323 (−0.515, −0.130)**
Characteristic path length (Lp)	0.79 ± 0.02	0.80 ± 0.02	419	0.323	−0.097 (−0.288, 0.095)
Normalized characteristic path length (λ)	0.47 ± 0.01	0.69 ± 0.08	419	<0.001*	**−0.423 (−0.616, −0.229)**
Small-worldness (σ)	0.67 ± 0.07	0.48 ± 0.02	419	0.008*	**−0.260 (−0.452, −0.068)**
Global efficiency (Eglob)	0.27 ± 0.01	0.27 ± 0.01	419	0.293	0.103 (−0.089, 0.294)
Local efficiency (Eloc)	0.34 ± 0.01	0.33 ± 0.01	419	<0.001*	**−0.365 (−0.557, −0.172)**

SCD, subjective cognitive decline; HC, healthy controls; df, degree of freedom; CI, confidence interval. *P-value < 0.05; The values in bold indicate *p*-values and their confidence intervals for indicators with statistical differences.

### 3.3 Comparison of topological properties in the high- and low- amplitude frame network between SCD and HC

**FIGURE 2:**
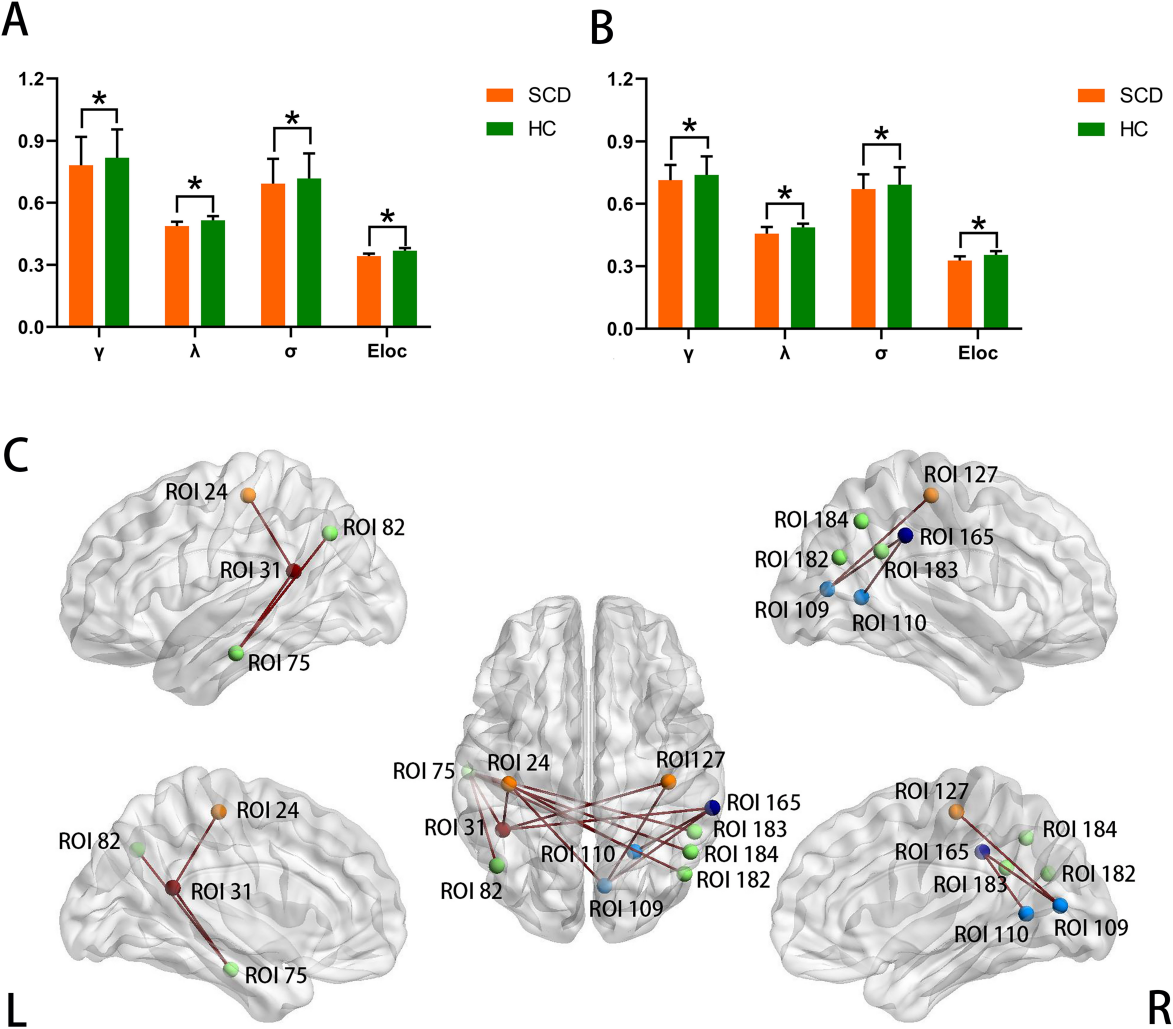
The graphs illustrate differences in network metrics between SCD and HC in high- and low-amplitude frame networks. In both high-amplitude **(A)** and low-amplitude **(B)** frame networks, significant reductions were found in γ, λ, σ, and Eloc in SCD. Asterisks (*) indicate significant between-group differences (*p* < 0.05). In high-amplitude frame network, panel **(C)** shows ROIs with significant differences in any nodal properties between two groups and connections with significant differences between these ROIs. Each ROI represents a brain region and each line represents a connection. Compared to HC, significantly increased connections in SCD are presented in dark red. Different-color ROIs denote different network: orange, somatomotor network; dark red, dorsal attention network; green, default mode network; lake blue, visual network; blue, frontoparietal task control network. ROI 24: a node in left postcentral gyrus; ROI 31: a node in left fusiform gyrus; ROI 75: a node in left inferior temporal gyrus; ROI 82: a node in left angular gyrus; ROI 109, ROI 110: nodes in right calcarine fissure and surrounding cortex; ROI 127: a node in right postcentral gyrus; ROI 165: a node in right supramarginal gyrus; ROI 182, ROI 183 and ROI 184: nodes in right angular gyrus. SCD, subjective cognitive decline; HC, healthy controls; γ, normalized clustering coefficient; λ, normalized characteristic path length; σ, small-worldness; Eloc, local efficiency.

In both high- and low-amplitude frame networks, the γ, λ, σ, and Eloc were significantly lower in SCD than that in HC (all *p* < 0.05; Figure 2 and Table 2). No statistically significant differences were observed in Cp, Lp and Eglob between the two groups. In the high-amplitude frame network, the betweenness centrality of the 10 ROIs in SCD was lower than that in HC, mainly involving the left postcentral gyrus, left inferior temporal gyrus, left angular gyrus, right calcarine fissure and surrounding cortex, right postcentral gyrus, right supramarginal gyrus and right angular gyrus; the nodal efficiency in the left fusiform gyrus was higher in SCD than in HC. In the low-amplitude frame networks, we found that the nodal centrality of 3 ROIs (left postcentral gyrus, left inferior temporal gyrus, right middle occipital gyrus) in SCD was significantly lower than that in HC (Table 3).

**TABLE 3:** Brain regions showing altered nodal centralities in SCD relative to HC.

Brain regions	SCD group (*n* = 211)	HC group (*n* = 210)	df	*P*-value	Cohen’s d (95% CI)
**High-amplitude frames network**					
Nodal betweenness in left postcentral gyrus (ROI 24)	31.79 ± 25.63	44.44 ± 37.90	419	<0.001*	**−0.391 (−0.584, −0.198)**
Nodal betweenness in left inferior temporal gyrus (ROI 75)	33.99 ± 34.61	45.24 ± 40.09	419	0.040*	**−0.301 (−0.493, −0.108)**
Nodal betweenness in left angular gyrus (ROI 82)	35.29 ± 28.90	48.00 ± 37.56	419	<0.001*	**−0.379 (−0.572, −0.186)**
Nodal betweenness in right calcarine fissure and surrounding cortex (ROI 109)	42.29 ± 27.30	54.63 ± 48.56	419	0.022*	**−0.314 (−0.506, −0.121)**
Nodal betweenness in right calcarine fissure and surrounding cortex (ROI 110)	30.88 ± 25.01	44.97 ± 39.96	419	<0.001*	**−0.423 (−0.616, −0.229)**
Nodal betweenness in right postcentral gyrus (ROI 127)	26.52 ± 25.89	37.10 ± 30.43	419	<0.001*	**−0.374 (−0.566, −0.181)**
Nodal betweenness in right supramarginal gyrus (ROI 165)	32.33 ± 28.29	43.96 ± 33.69	419	<0.001*	**−0.375 (−0.567, −0.183)**
Nodal betweenness in right angular gyrus (ROI 182)	39.32 ± 34.62	54.04 ± 39.62	419	<0.001*	**−0.396 (−0.588, −0.203)**
Nodal betweenness in right angular gyrus (ROI 183)	50.14 ± 34.57	64.97 ± 45.31	419	<0.001*	**−0.368 (−0.561, −0.175)**
Nodal betweenness in right angular gyrus (ROI 184)	27.75 ± 26.15	39.49 ± 30.36	419	<0.001*	**−0.415 (−0.607, −0.221)**
Nodal efficiency in left fusiform gyrus (ROI 31)	0.28 ± 0.03	0.27 ± 0.03	419	<0.001*	**0.347 (0.154, 0.539)**
**Low-amplitude frames network**					
Nodal betweenness in left inferior temporal gyrus (ROI 75)	32.49 ± 27.57	42.41 ± 28.65	419	<0.001*	**−0.353 (−0.545, −0.160)**
Nodal efficiency in left postcentral gyrus (ROI 33)	0.27 ± 0.04	0.28 ± 0.03	419	<0.001*	**−0.356 (−0.548, −0.163)**
Nodal efficiency in right middle occipital gyrus (ROI 115)	0.27 ± 0.03	0.28 ± 0.03	419	<0.001*	**−0.347 (−0.540, −0.155)**
Nodal degree in right middle occipital gyrus (ROI 115)	25.06 ± 9.17	28.12 ± 8.43	419	<0.001*	**−0.348 (−0.540, −0.155)**

SCD, subjective cognitive decline; HC, healthy controls; df, degree of freedom; CI, confidence interval; ROI, regions of interest. The abnormal brain regions were determined if at least one of the three nodal centralities showed a significant between-group difference (*P* < 0.05, FDR corrected). *P-value < 0.05, FDR corrected. The values in bold indicate *P*-values and their confidence intervals for indicators with statistical differences.

### 3.4 SCD-related subnetworks

In the NBS analysis of high-amplitude frame networks, a subnetwork with significantly increased functional connectivity was identified in SCD. This subnetwork consisted of 11 ROIs and 13 connections, primarily involving the default mode network (DMN) (Figure 2). In contrast, the NBS analysis of low-amplitude frame networks revealed no statistically significant differences in connections between the two groups.

### 3.5 Correlation between network metrics and clinical variables

**FIGURE 3:**
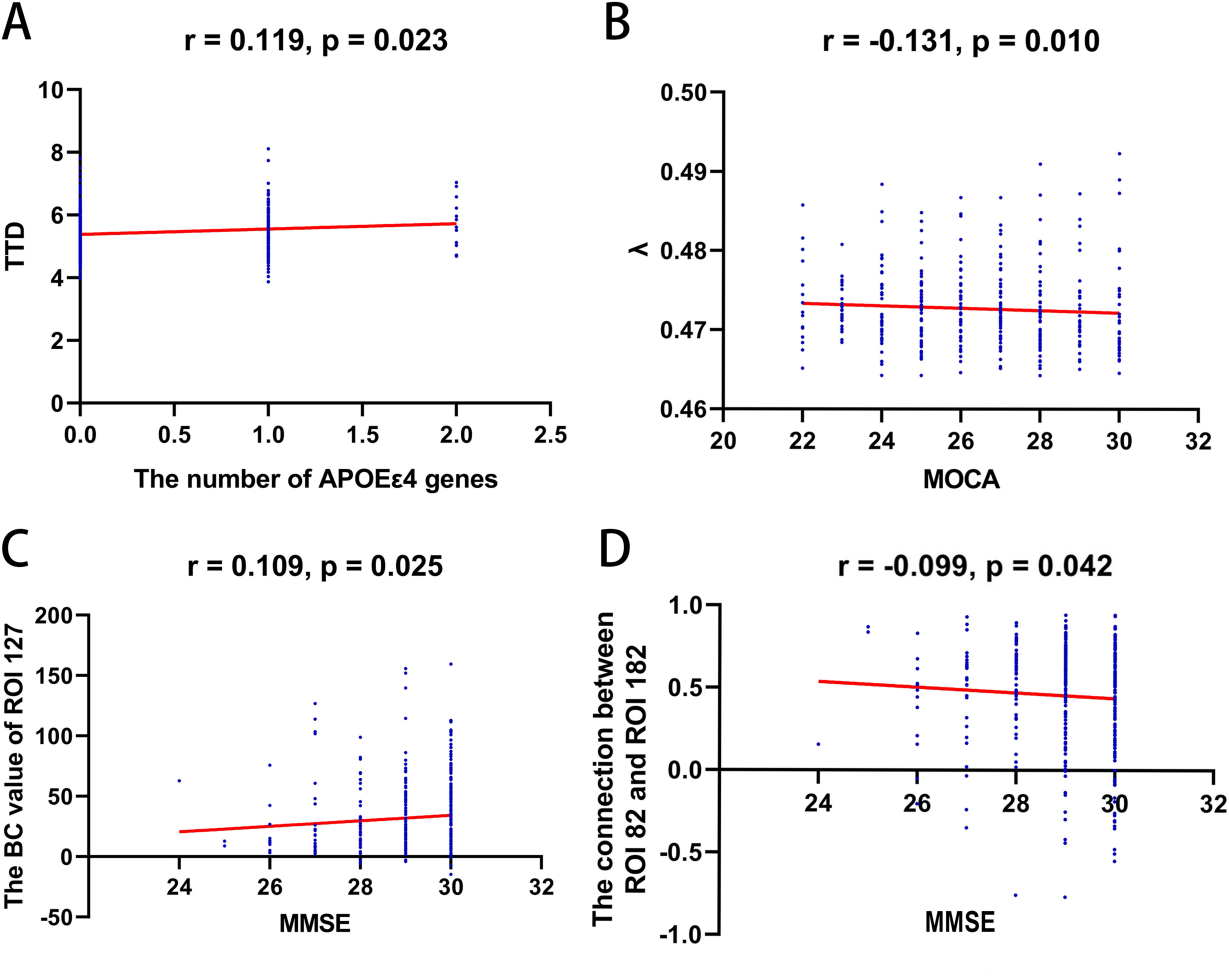
Correlations between TTD, network metrics, cognitive scores, and the number of APOE ε4 genes. **(A)** Correlation between TTD and the number of APOE ε4 genes (*p* = 0.023, *r* = 0.119). **(B)** Correlation between λ in low-amplitude frame network and MOCA (*p* = 0.010, *r* = –0.131). **(C,D)** In the high-amplitude frame network, the BC value of ROI 127 was significantly positively correlated with MMSE (*p* = 0.025, *r* = 0.109). The connection between ROI 82 and ROI 182 was negatively correlated with MMSE (*p* = 0.042, *r* = –0.099). ROI 82: a node in left angular gyrus; ROI 127: a node in right postcentral gyrus; ROI 182: a node in right angular gyrus. TTD, duration of trough-to-trough; APOE-ε 4, apolipoprotein E ε 4; λ, normalized characteristic path length; Bc, nodal betweenness centrality; MOCA, the Montreal Cognitive Assessment; MMSE, Mini-Mental State Examination.

There was a significant positive correlation between the number of APOE ε4 genes and TTD (*r* = 0.119, *p* = 0.023). In low-amplitude frame network, the λ was negatively correlated with MOCA (*r* = −0.131, *p* = 0.010). In high-amplitude frame network, the betweenness centrality of ROI 127 (right postcentral gyrus) was positively related to MMSE (*r* = 0.109, *p* = 0.025). However, the connection between ROI 82 (left angular gyrus) and ROI 182 (right angular gyrus) exhibited a significant negative correlation with MMSE (*r* = −0.099, *p* = 0.042). No significant associations were found between other network metrics and clinical variables (Figure 3).

## 4 Discussion

In this study, we constructed high- and low-amplitude frame networks through an edge-centric FC framework to distinguish SCD from HC. The results revealed that SCD patients exhibited significantly lower average peak amplitude and longer trough-to-trough duration (TTD) compared to HC, with TTD showing a positive correlation with the number of APOE ε4 genes. In both high- and low-amplitude frame networks, the global and nodal topology indicators of SCD primarily exhibited a decreasing trend. However, high-amplitude frame networks demonstrated more altered nodal centrality and SCD-related subnetworks. Furthermore, some altered topological indicators were correlated with cognitive scores.

Traditional node-centric functional connectivity (nFC) analysis is often used to reveal abnormal FC or disease classification in brain diseases. In nFC analysis, the assessment of correlation reflects the interactive information between two brain regions in brain space. However, there are often interactions between multiple brain regions in the brain, and these more complex interactions may not be captured in nFC (Zhao et al., 2018). Recently, Faskowitz et al. (2020) proposed an edge-to-edge network framework for highlighting interactions between edges. In eFC analysis, the measure of correlation corresponds to the co-fluctuation similarity between edges, which can be understood as a “talk” between pairs of brain regions. This involves higher-order interactions among the four brain regions. Therefore, eFC highlights the unique features of different levels in the brain networks compared to nFC. Our research reveals the utility of eFC analysis in localizing brain regions closely linked to SCD. These findings may lay the foundation for building the diagnostic frameworks of SCD in the future.

In our study, the mean peak amplitude, which reflects the intensity of the brain’s BOLD signal, was found to correlate with the degree of brain activity. The results show that compared with HC, the mean peak amplitude of SCD is significantly reduced, indicating a weakening of brain network activity in SCD. SCD is considered as a pre-clinical stage of AD, which has similar pathophysiological changes with AD, for example, Aβ and tau protein deposition. It has been suggested that changes in neuronal electrical activity and network oscillations are one of the first signals in the brain of AD patients before the onset of clinical symptoms, and they are closely related to Aβ deposition (Harris et al., 2020; Palop and Mucke, 2016). The abnormal accumulation of Aβ in the brain exerts adverse effects on neurons, such as inducing neuritic plaques formation, disrupting calcium homeostasis, synaptic loss, and oxidative stress, thereby directly leading to neuronal dysfunction and death (Spires-Jones and Hyman, 2014). Melo de Farias et al. (2023) found that a slight elevation in Aβ concentration are sufficient to cause transcriptional changes in human neurons that contribute to early alterations in neural network activity. Based on the above, we speculate that reduced brain network activity in SCD may be potentially related to neuronal dysfunction and death resulting from pathophysiological processes associated with AD spectrum disorders. Moreover, a strong relationship exists between flexible brain dynamics and cognition (Lee et al., 2022). To adapt to new cognitive demands, the maintenance of brain state transition function is crucial for proper information processing and resource reconfiguration (Lee et al., 2022; Taghia et al., 2018). TTD represents the time interval required for brain state transitions, with a shorter TTD indicating greater brain flexibility. In our study, the longer TTD observed in SCD compared to HC suggests that dynamic brain networks in SCD may be more blunted and slower, resulting in delayed or blocked state transitions. Consequently, during continuous cognitive processes, the time required for SCD patients will be prolonged (Liu W. et al., 2021; Varangis et al., 2022). It is noteworthy that we observed a significant positive correlation between TTD and the number of APOE ε4 genes. The APOE ε4 gene is the strongest risk factor for AD, and the severity of cognitive impairment correlates with the number of APOE ε4 genes in a dose-dependent manner (Makkar et al., 2020; Wang et al., 2021). In general, the apolipoprotein E4 (encoded by APOE ε4 genes) is implicated in the pathogenesis of AD by promoting cerebral Aβ deposition and accelerating tau hyperphosphorylation and tangle formation through various molecular mechanisms that underlie the deterioration of structural and functional networks (Bilousova et al., 2019; Raulin et al., 2024). Early studies have shown that Aβ can damage the function of all cell types comprising the neurovascular unit and some inhibitory interneurons, resulting in the reduction of neurovascular coupling efficiency, disruption of the excitation/inhibition balance within brain networks, and impaired network synchronization (Iadecola, 2004; Palop and Mucke, 2016). These alterations collectively diminish the overall quality and efficiency of brain network connectivity. Another study showed that elevated plasma p-tau231 was related to reduced dynamic network flexibility in the medial temporal lobe among healthy older African Americans (Budak et al., 2024). In conclusion, we speculate that the development of Aβ and tau pathology may be related to the changes in brain dynamics of AD continuum, and these processes may be regulated or influenced by APOE ε4 genes. In our study, TTD can be regarded as an indicator of the flexibility of brain network state transition, and its correlation with APOE ε4 genes provides clues for future research. Further work is needed to elucidate the interplay between TTD alterations and Aβ/tau pathology along the AD continuum, and to determine whether the APOE ε4 genes moderates these associations. Furthermore, based on the fundamental nature of this study as an exploratory study, the primary objective is to investigate the potential value of edge-centric networks in identifying SCD. To avoid prematurely excluding meaningful cues, our correlation analysis did not employ multiple comparison correction. We acknowledge that this practice increases the likelihood of finding a false positive association. Therefore, we recommend that these associations be considered preliminary results and need to be replicated for validation in larger studies.

Another important finding in our study showed that the difference between high- and low-amplitude frame networks in discriminating SCD and HC existed at the nodal level. A total of 11 ROIs were detected to have altered nodal centrality in the high-amplitude frame network, whereas only 3 ROIs were found to have decreased nodal centrality in the low-amplitude frame network. As is well known, a wide range of brain regions are associated with social behaviors and memory processes, and the coordination of neurons ensembles within and across these regions is essential for supporting complex cognitive functions (Oliva, 2023). Previous studies have indicated that higher co-fluctuations across the brain reflect higher overall brain synchronization; conversely, lower co-fluctuations represent weaker brain synchronization (Faskowitz et al., 2020; Sasse et al., 2023). To some extent, the high synchronicity of the brain leads to a strong integration state among functional networks, which is associated with better cognitive performance (Grover et al., 2021; Shine et al., 2016). Therefore, the observed differences in nodal centrality across more ROIs in the high-amplitude frame network can be attributed to the strong integration state of the brain, which recruits more brain regions to participate in cognitive processes.

Additionally, in both high and low-amplitude frame networks, the reduced nodal centrality (Bc and Dc) in SCD was primarily distributed in the DMN. Large-scale network disruption in the DMN is the earliest alteration associated with AD (Lee et al., 2023; Sharma et al., 2021). During the SCD period, not only the functional network, but also anatomical and morphological network features were predominantly located in the DMN (Chen et al., 2022). At the same time, we found an SCD-related subnetwork in the high-amplitude frame network, which consists of 11 ROIs and 13 edges. The enhanced FC within this subnetwork reflects the internal hypersynchronous state. It remains to be verified whether this is associated with the disruption of excitation-inhibition balance in key regions (e.g., the default mode network) caused by early pathological changes. Regarding changes in the global metrics of SCD, we found that the global metrics (γ, λ, σ and Eloc) of SCD exhibited a decreasing trend in both high- and low-amplitude frame networks. In detail, the decreased segregation (lower γ and Eloc) and increased integration (lower λ) of SCD reflect the alterations of functional randomization in brain networks (Suo et al., 2018). Previous studies have reported similar results, showing decreased γ, σ and Eloc in SCD, with these alterations will become more significant as cognitive impairment worsens (Li et al., 2021; Liu Y. et al., 2021; Zhang et al., 2023). We speculated that these changes may result from neurodegeneration. In conclusion, the weakening, interruption, or recombination of connections in brain networks may be a critical neural substrate underlying the pathological damage associated with SCD.

Our study demonstrates distinct patterns of FC under the edge-centric network framework between SCD and HC. However, it should be noted that the effect sizes for these differences were small. This observation is conceptually consistent with the understanding that SCD represents a prodromal stage of the AD continuum, where neuropathological changes are incipient and neural network disruption is inherently subtle. Both SCD and HC groups performed within the normal range on objective cognitive tests. The inherent similarities between these two groups may have limited the magnitude of observable differences in network measures. Nevertheless, the fact that our edge-centric method was able to detect these statistically robust, albeit subtle, changes highlights its sensitivity in exploring early neural alterations associated with cognition. From a clinical perspective, it is critical to identify such subtle differences, as they may constitute the earliest precursors to subsequent network disruption. Future longitudinal studies should prospectively investigate the ability of these subtle network alterations to predict disease progression and track whether their effect sizes are amplified throughout its course.

## 5 Limitation

The limitations of this study are as follows: (1) as a cross-sectional study, it lacks dynamic follow-up analysis. Future longitudinal studies should incorporate tracking analyses across different stages of AD to provide more reliable information on the progression of AD-related functional networks. (2) The present study investigated the abnormalities of FC in SCD only in the resting state; future studies should validate these results under task-state fMRI. (3) At present, there is no unified standard for SCD, which is mainly judged by subjective feelings. The definition of SCD requires normal objective cognitive tests, but some patients may fail to detect mild cognitive impairment at an early stage due to high knowledge reserve or insufficient test sensitivity. In addition, cognitive changes can be caused by a variety of causes, including normal aging, emotional problems (anxiety, depression), chronic diseases, etc. Therefore, there may be heterogeneity in the selection of SCD samples. (4) Our study did not account for all potential confounders, such as the impact of cerebral small vessel disease (CSVD). CSVD-related structural changes can impair the integrity of both structural and functional networks, undermining efficient communication across the brain. White matter hyperintensities are associated with the destruction of structural white matter integrity. The damage of white matter fiber tracts makes the “path” of communication between brain regions longer or circuitous, which consequently compromises the brain network’s capacity for information integration and reduces efficiency in processing complex cognitive tasks (Ter Telgte et al., 2018). Similarly, variables related to cardiovascular diseases (e.g., hypertension, blood lipids, glucose, etc.) and lifestyle factors such as smoking may also modulate brain connectivity independently (Hazelton et al., 2025). Due to data accessibility, our study may not be able to fully strip away their effects. Consequently, our findings should be interpreted as preliminary evidence, and future research incorporating these covariates is necessary to elucidate the intrinsic associations more accurately.

## 6 Conclusion

Our study revealed that alterations of co-fluctuation between the edges in SCD brain networks, which may reflect important information about SCD-related neural substrates. Among them, TTD showed significant differences between groups and was related to APOE ε4 genes, which is expected to become a potential biomarker to assist in disease diagnosis. Furthermore, compared to low-amplitude frames, high-amplitude frames may better reflect changes in cognitive function. Collectively, as a promising supplementary tool, eFC may facilitate future research on the early diagnosis of the AD spectrum disorders.

## Data Availability

The datasets presented in this study can be found in online repositories. The names of the repository/repositories and accession number(s) can be found below: http://adni.loni.usc.edu/methods/documents/.

## Footnotes

1
https://adni.loni.usc.edu/

2
http://adni.loni.usc.edu/methods/documents/

## References

[B1] Behrens T. E. Sporns O. (2012). Human connectomics. *Curr. Opin. Neurobiol.* 22 144–153. 10.1016/j.conb.2011.08.005 21908183 PMC3294015

[B2] Bilousova T. Melnik M. Miyoshi E. Gonzalez B. L. Poon W. W. Vinters H. V. (2019). Apolipoprotein E/Amyloid-β complex accumulates in Alzheimer disease cortical synapses via apolipoprotein e receptors and is enhanced by APOE4. *Am. J. Pathol.* 189 1621–1636. 10.1016/j.ajpath.2019.04.010 31108099 PMC6680253

[B3] Blamire A. M. (2018). MR approaches in neurodegenerative disorders. *Prog. Nucl. Magn. Reson. Spectrosc.* 108 1–16. 10.1016/j.pnmrs.2018.11.001 30538047

[B4] Bolton C. J. Steinbach M. Khan O. A. Liu D. O’Malley J. Dumitrescu L. (2024). Clinical and demographic factors modify the association between plasma phosphorylated tau-181 and cognition. *Alzheimers Dement.* 16:e70047. 10.1002/dad2.70047 39713247 PMC11659951

[B5] Budak M. Fausto B. A. Osiecka Z. Sheikh M. Perna R. Ashton N. (2024). Elevated plasma p-tau231 is associated with reduced generalization and medial temporal lobe dynamic network flexibility among healthy older African Americans. *Alzheimers Res. Ther.* 16:253. 10.1186/s13195-024-01619-0 39578853 PMC11583385

[B6] Chen H. Li W. Sheng X. Ye Q. Zhao H. Xu Y. (2022). Machine learning based on the multimodal connectome can predict the preclinical stage of Alzheimer’s disease: A preliminary study. *Eur. Radiol.* 32 448–459. 10.1007/s00330-021-08080-9 34109489

[B8] Chen Q. Lu J. Zhang X. Sun Y. Chen W. Li X. (2021). Alterations in dynamic functional connectivity in individuals with subjective cognitive decline. *Front. Aging Neurosci.* 13:646017. 10.3389/fnagi.2021.646017 33613274 PMC7886811

[B9] Cohen J. R. (2018). The behavioral and cognitive relevance of time-varying, dynamic changes in functional connectivity. *NeuroImage* 180 515–525. 10.1016/j.neuroimage.2017.09.036 28942061 PMC6056319

[B10] Faskowitz J. Esfahlani F. Z. Jo Y. Sporns O. Betzel R. F. (2020). Edge-centric functional network representations of human cerebral cortex reveal overlapping system-level architecture. *Nat. Neurosci.* 23 1644–1654. 10.1038/s41593-020-00719-y 33077948

[B11] Gao R. van den Brink R. L. Pfeffer T. Voytek B. (2020). Neuronal timescales are functionally dynamic and shaped by cortical microarchitecture. *eLife* 9:e61277. 10.7554/eLife.61277 33226336 PMC7755395

[B12] Grover S. Nguyen J. A. Reinhart R. M. G. (2021). Synchronizing brain rhythms to improve cognition. *Annu. Rev. Med.* 72 29–43. 10.1146/annurev-med-060619-022857 33035432 PMC10068593

[B13] Harris S. S. Wolf F. De Strooper B. Busche M. A. (2020). Tipping the scales: Peptide-dependent dysregulation of neural circuit dynamics in Alzheimer’s disease. *Neuron* 107 417–435. 10.1016/j.neuron.2020.06.005 32579881

[B14] Hazelton J. L. Migeot J. Gonzalez-Gomez R. Altschuler F. Duran-Aniotz C. Wen O. (2025). Cardiovascular risk factors and the allostatic interoceptive network in dementia. *Cardiovasc. Res.* 121 2222–2232. 10.1093/cvr/cvaf185 41073365 PMC12638725

[B15] Iadecola C. (2004). Neurovascular regulation in the normal brain and in Alzheimer’s disease. *Nat. Rev. Neurosci.* 5 347–360. 10.1038/nrn1387 15100718

[B16] Idesis S. Faskowitz J. Betzel R. F. Corbetta M. Sporns O. Deco G. (2022). Edge-centric analysis of stroke patients: An alternative approach for biomarkers of lesion recovery. *Neuroimage Clin.* 35:103055. 10.1016/j.nicl.2022.103055 35661469 PMC9163596

[B17] Jessen F. Amariglio R. E. Buckley R. F. van der Flier W. M. Han Y. Molinuevo J. L. (2020). The characterisation of subjective cognitive decline. *Lancet Neurol.* 19 271–278. 10.1016/s1474-4422(19)30368-0 31958406 PMC7062546

[B18] Jessen F. Amariglio R. E. van Boxtel M. Breteler M. Ceccaldi M. Chételat G. (2014). A conceptual framework for research on subjective cognitive decline in preclinical Alzheimer’s disease. *Alzheimers Dement.* 10 844–852. 10.1016/j.jalz.2014.01.001 24798886 PMC4317324

[B19] Jiang W. Merhar S. L. Zeng Z. Zhu Z. Yin W. Zhou Z. (2022). Neural alterations in opioid-exposed infants revealed by edge-centric brain functional networks. *Brain Commun.* 4:fcac112. 10.1093/braincomms/fcac112 35602654 PMC9117006

[B20] Kang M. Li C. Mahajan A. Spat-Lemus J. Durape S. Chen J. (2024). Subjective cognitive decline plus and longitudinal assessment and risk for cognitive impairment. *JAMA Psychiatry* 81 993–1002. 10.1001/jamapsychiatry.2024.1678 38959008 PMC11223054

[B21] Lee B. Cai W. Young C. B. Yuan R. Ryman S. Kim J. (2022). Latent brain state dynamics and cognitive flexibility in older adults. *Prog. Neurobiol.* 208:102180. 10.1016/j.pneurobio.2021.102180 34627994 PMC9585912

[B22] Lee D. Park J. Y. Kim W. J. (2023). Altered functional connectivity of the default mode and dorsal attention network in subjective cognitive decline. *J. Psychiatr. Res.* 159 165–171. 10.1016/j.jpsychires.2023.01.040 36738647

[B24] Li Z. Han Y. Jiang J. (2021). Different brain functional networks between subjective cognitive decline and health control based on graph theory. *Annu. Int. Conf. IEEE Eng. Med. Biol. Soc.* 2021 5752–5755. 10.1109/embc46164.2021.9630421 34892426

[B25] Liew T. M. (2020). Trajectories of subjective cognitive decline, and the risk of mild cognitive impairment and dementia. *Alzheimers Res. Ther.* 12:135. 10.1186/s13195-020-00699-y 33109275 PMC7592368

[B26] Ligero M. Jordi-Ollero O. Bernatowicz K. Garcia-Ruiz A. Delgado-Muñoz E. Leiva D. (2021). Minimizing acquisition-related radiomics variability by image resampling and batch effect correction to allow for large-scale data analysis. *Eur. Radiol.* 31 1460–1470. 10.1007/s00330-020-07174-0 32909055 PMC7880962

[B27] Lin Y. Shan P. Y. Jiang W. J. Sheng C. Ma L. (2019). Subjective cognitive decline: Preclinical manifestation of Alzheimer’s disease. *Neurol. Sci.* 40 41–49. 10.1007/s10072-018-3620-y 30397816

[B28] Liu W. Kohn N. Fernández G. (2021). Dynamic transitions between neural states are associated with flexible task switching during a memory task. *J. Cogn. Neurosci.* 33 2559–2588. 10.1162/jocn_a_01779 34644388

[B29] Liu Y. Li Z. Jiang X. Du W. Wang X. Sheng C. (2021). Differences in functional brain networks between subjective cognitive decline with and without worry groups: A graph theory study from SILCODE. *J. Alzheimers Dis.* 84 1279–1289. 10.3233/jad-215156 34657889

[B30] Makkar S. R. Lipnicki D. M. Crawford J. D. Kochan N. A. Castro-Costa E. Lima-Costa M. F. (2020). APOE ε4 and the influence of sex, age, vascular risk factors, and ethnicity on cognitive decline. *J. Gerontol. A Biol. Sci. Med. Sci.* 75 1863–1873. 10.1093/gerona/glaa116 32396611 PMC7518559

[B31] Melo de Farias A. R. Pelletier A. Iohan L. C. C. Saha O. Bonnefond A. (2023). Amyloid-Beta peptides trigger premature functional and gene expression alterations in human-induced neurons. *Biomedicines* 11:2564. 10.3390/biomedicines11092564 37761004 PMC10526858

[B32] Oliva A. (2023). Neuronal ensemble dynamics in social memory. *Curr. Opin. Neurobiol.* 78:102654. 10.1016/j.conb.2022.102654 36509026

[B33] Palop J. J. Mucke L. (2016). Network abnormalities and interneuron dysfunction in Alzheimer disease. *Nat. Rev. Neurosci.* 17 777–792. 10.1038/nrn.2016.141 27829687 PMC8162106

[B34] Pérez-Cordón A. Monté-Rubio G. Sanabria A. Rodriguez-Gomez O. Valero S. Abdelnour C. (2020). Subtle executive deficits are associated with higher brain amyloid burden and lower cortical volume in subjective cognitive decline: The FACEHBI cohort. *Sci. Rep.* 10:17721. 10.1038/s41598-020-74704-7 33082443 PMC7576802

[B35] Pike K. E. Cavuoto M. G. Li L. Wright B. J. Kinsella G. J. (2022). Subjective cognitive decline: Level of risk for future dementia and mild cognitive impairment, a meta-analysis of longitudinal studies. *Neuropsychol. Rev.* 32 703–735. 10.1007/s11065-021-09522-3 34748154

[B36] Raulin A. C. Doss S. V. Heckman M. G. Craver E. C. Li Z. Ikezu T. C. (2024). Impact of APOE on amyloid and tau accumulation in argyrophilic grain disease and Alzheimer’s disease. *Acta Neuropathol. Commun.* 12:25. 10.1186/s40478-024-01731-0 38336940 PMC10854035

[B37] Risacher S. L. Saykin A. J. (2019). Neuroimaging in aging and neurologic diseases. *Handb. Clin. Neurol.* 167 191–227. 10.1016/b978-0-12-804766-8.00012-1 31753134 PMC9006168

[B38] Sasse L. Larabi D. I. Omidvarnia A. Jung K. Hoffstaedter F. Jocham G. (2023). Intermediately synchronised brain states optimise trade-off between subject specificity and predictive capacity. *Commun. Biol.* 6:705. 10.1038/s42003-023-05073-w 37429937 PMC10333234

[B39] Schaefer A. Kong R. Gordon E. M. Laumann T. O. Zuo X. N. Holmes A. J. (2018). Local-Global parcellation of the human cerebral cortex from intrinsic functional connectivity MRI. *Cereb. Cortex* 28 3095–3114. 10.1093/cercor/bhx179 28981612 PMC6095216

[B40] Serra L. Bonarota S. Di Domenico C. Caruso G. Giulietti G. Caltagirone C. (2023). Preclinical brain network abnormalities in patients with subjective cognitive decline. *J. Alzheimers Dis.* 95 1119–1131. 10.3233/jad-230536 37661886

[B41] Sharma N. Murari G. Vandermorris S. Verhoeff N. Herrmann N. Chen J. J. (2021). Functional connectivity between the posterior default mode network and parahippocampal gyrus is disrupted in older adults with subjective cognitive decline and correlates with subjective memory ability. *J. Alzheimers Dis.* 82 435–445. 10.3233/jad-201579 34024823

[B42] Shine J. M. Bissett P. G. Bell P. T. Koyejo O. Balsters J. H. Gorgolewski K. J. (2016). The dynamics of functional brain networks: Integrated network states during cognitive task performance. *Neuron* 92 544–554. 10.1016/j.neuron.2016.09.018 27693256 PMC5073034

[B43] Spires-Jones T. L. Hyman B. T. (2014). The intersection of amyloid beta and tau at synapses in Alzheimer’s disease. *Neuron* 82 756–771. 10.1016/j.neuron.2014.05.004 24853936 PMC4135182

[B44] Sporns O. Faskowitz J. Teixeira A. S. Cutts S. A. Betzel R. F. (2021). Dynamic expression of brain functional systems disclosed by fine-scale analysis of edge time series. *Netw. Neurosci.* 5 405–433. 10.1162/netn_a_00182 34189371 PMC8233118

[B45] Sun A. Wang J. Zhang J. (2023). Identifying autism spectrum disorder using edge-centric functional connectivity. *Cereb. Cortex* 33 8122–8130. 10.1093/cercor/bhad103 36977635

[B46] Suo X. S. Lei D. L. Li L. L. Li W. L. Dai J. D. Wang S. W. (2018). Psychoradiological patterns of small-world properties and a systematic review of connectome studies of patients with 6 major psychiatric disorders. *J. Psychiatry Neurosci.* 43:427. 10.1503/jpn.170214 30375837 PMC6203546

[B47] Taghia J. Cai W. Ryali S. Kochalka J. Nicholas J. Chen T. (2018). Uncovering hidden brain state dynamics that regulate performance and decision-making during cognition. *Nat. Commun.* 9:2505. 10.1038/s41467-018-04723-6 29950686 PMC6021386

[B48] Ter Telgte A. van Leijsen E. M. C. Wiegertjes K. Klijn C. J. M. Tuladhar A. M. de Leeuw F. E. (2018). Cerebral small vessel disease: From a focal to a global perspective. *Nat. Rev. Neurol.* 14 387–398. 10.1038/s41582-018-0014-y 29802354

[B49] Thiebaut de Schotten M. Forkel S. J. (2022). The emergent properties of the connected brain. *Science* 378 505–510. 10.1126/science.abq2591 36378968

[B50] Varangis E. Qi W. Stern Y. Lee S. (2022). The role of neural flexibility in cognitive aging. *Neuroimage* 247:118784. 10.1016/j.neuroimage.2021.118784 34902547 PMC9055953

[B51] Wang G. Vance D. E. Li W. (2021). A cross-sectional analysis of APOE gene polymorphism and the risk of cognitive impairments in the Alzheimer’s disease neuroimaging initiative study. *JAR Life* 10 26–31. 10.14283/jarlife.2021.5 36923510 PMC10002875

[B52] Wang Q. Chen B. Zhong X. Hou L. Zhang M. Yang M. (2022). Static and dynamic functional connectivity variability of the anterior-posterior hippocampus with subjective cognitive decline. *Alzheimers Res. Ther.* 14:122. 10.1186/s13195-022-01066-9 36057586 PMC9440588

[B53] Wang X. Huang W. Su L. Xing Y. Jessen F. Sun Y. (2020). Neuroimaging advances regarding subjective cognitive decline in preclinical Alzheimer’s disease. *Mol. Neurodegener.* 15:55. 10.1186/s13024-020-00395-3 32962744 PMC7507636

[B54] Watts D. J. Strogatz S. H. (1998). Collective dynamics of ‘small-world’ networks. *Nature* 393 440–442. 10.1038/30918 9623998

[B55] Zamani Esfahlani F. Byrge L. Tanner J. Sporns O. Kennedy D. P. Betzel R. F. (2022). Edge-centric analysis of time-varying functional brain networks with applications in autism spectrum disorder. *Neuroimage* 263:119591. 10.1016/j.neuroimage.2022.119591 36031181 PMC12403185

[B56] Zhang H. Q. Chau A. C. M. Shea Y. F. Chiu P. K. Bao Y. W. Cao P. (2023). Disrupted structural white matter network in Alzheimer’s disease continuum, vascular dementia, and mixed dementia: A diffusion tensor imaging study. *J. Alzheimers Dis.* 94 1487–1502. 10.3233/jad-230341 37424470

[B57] Zhao F. Zhang H. Rekik I. An Z. Shen D. J. (2018). Diagnosis of autism spectrum disorders using multi-level high-order functional networks derived from resting-state functional MRI. *Front. Hum. Neurosci.* 12:184. 10.3389/fnhum.2018.00184 29867410 PMC5960713

