# Supplementary material for: Altered brain network dynamics and functional connectivity in subjective cognitive decline: an edge-centric network study
Source: Front Aging Neurosci. 2026 Jan 9;17:1596537. doi: 10.3389/fnagi.2025.1596537 (PMC12827695; doi:10.3389/fnagi.2025.1596537)
Supplement: Supplementary file 1 [file Data_Sheet_1.pdf]

Supplementary material

1. fMRI data acquisition

A total of 421 subjects were enrolled, of which 211 subjective cognitive decline (SCD) patients were scanned using 14 scanners, while the health controls (HC) was scanned using 13 scanners. There were slight differences in the scanning parameters of different scanners.

Regarding the SCD group, the parameters of fMRI images acquired by scanner 1 (Siemens Magnetom, Prisma) were as follows: repetition time (TR)/echo time (TE) = 3000ms/30ms, matrix = 448 × 448, voxel size = 3.4 mm × 3.4 mm, flip angle = 90, slices = 197, slice thickness = 3.4 mm.

The parameters of fMRI images acquired by scanner 2 (Siemens Magnetom, Prisma) were as follows: TR/TE = 607ms/32ms, matrix = 704 × 704, voxel size = 2.5 mm × 2.5mm, flip angle = 50, slices = 976, slice thickness = 2.5 mm.

The parameters of fMRI images acquired by scanner 3 (Siemens Magnetom, Skyra) were as follows: TR/TE = 3400ms/13ms, matrix = 320 × 320, voxel size = 3.8 mm × 3.8mm, flip angle = 90, slices = 105, slice thickness = 4.0 mm.

The parameters of fMRI images acquired by scanner 4 (Siemens Magnetom, TrioTim) were as follows: TR/TE = 3400ms/12ms, matrix = 320 × 320, voxel size = 4.0 mm × 4.0mm, flip angle = 90, slices = 105, slice thickness = 4.0 mm.

The parameters of fMRI images acquired by scanner 5 (Siemens Magnetom, TrioTim) were as follows: TR/TE = 3000ms/30ms, matrix = 448 × 448, voxel size = 3.4 mm × 3.4mm, flip angle = 90, slices = 197, slice thickness = 3.4 mm.

The parameters of fMRI images acquired by scanner 6 (Siemens Magnetom, Verio) were as follows: TR/TE = 3400ms/13ms, matrix = 320 × 320, voxel size = 4.0 mm × 4.0mm, flip angle = 90, slices = 105, slice thickness

= 4.0 mm.

The parameters of fMRI images acquired by scanner 7 (Siemens Magnetom, Verio) were as follows: TR/TE = 3000ms/30ms, matrix = 448 × 448, voxel size = 3.4 mm × 3.4mm, flip angle = 90, slices = 197, slice thickness = 3.4 mm.

The parameters of fMRI images acquired by scanner 8 (GE MEDICAL SYSTEMS, DISCOVERY MR750) were as follows: TR/TE = 3000ms/30ms, matrix = 64 × 64, voxel size = 3.4 mm × 3.4mm, flip angle = 90, slices = 9600, slice thickness = 3.4 mm.

The parameters of fMRI images acquired by scanner 9 (GE MEDICAL SYSTEMS, SIGNA UHP) were as follows: TR/TE = 3000ms/30ms, matrix = 64 × 64, voxel size = 3.4 mm × 3.4mm, flip angle = 90, slices = 9600, slice thickness = 3.4 mm.

The parameters of fMRI images acquired by scanner 10 (Philips Healthcare, Ingenia) were as follows: TR/TE = 3000ms/30ms, matrix = 64 × 64, voxel size = 3.4 mm × 3.4mm, flip angle = 90, slices = 9456, slice thickness = 3.4 mm.

The parameters of fMRI images acquired by scanner 11 (Philips Medical Systems, Ingenuity) were as follows: TR/TE = 3000ms/30ms, matrix = 64 × 64, voxel size = 3.3 mm × 3.3mm, flip angle = 80, slices = 6720, slice thickness = 3.3 mm.

The parameters of fMRI images acquired by scanner 12 (Philips Medical Systems, Intera) were as follows: TR/TE = 3001ms/30ms, matrix = 64 × 64, voxel size = 3.3 mm × 3.3mm, flip angle = 80, slices = 6720, slice thickness = 3.3 mm.

The parameters of fMRI images acquired by scanner 13 (Philips Medical Systems, Achieva) were as follows: TR/TE = 3000ms/30ms, matrix = 64 × 64, voxel size = 3.3 mm × 3.3mm, flip angle = 80, slices = 6720, slice thickness = 3.3 mm.

The parameters of fMRI images acquired by scanner 14 (Philips Medical Systems, Achieva) were as follows: TR/TE = 3000ms/30ms, matrix = 64 × 64, voxel size = 3.4 mm × 3.4mm, flip angle = 90, slices = 9456, slice thickness = 3.4 mm.

For the HC group, the scanner models and imaging parameters for scanners 1 to 8, 10, and 13 were consistent with those utilized in the SCD group.

The parameters of fMRI images acquired by scanner 9 (GE MEDICAL SYSTEMS, SIGNA Premier) were as follows: TR/TE = 3000ms/30ms, matrix = 64 × 64, voxel size = 3.4 mm × 3.4mm, flip angle = 90, slices = 9600, slice thickness = 3.4 mm.

The parameters of fMRI images acquired by scanner 11 (Philips Healthcare, Ingenia) were as follows: TR/TE = 3000ms/30ms, matrix = 64 × 64, voxel size = 3.3 mm × 3.3mm, flip angle = 80, slices = 6720, slice thickness = 3.3mm.

The parameters of fMRI images acquired by scanner 12 (Philips Medical Systems, Intera) were as follows: TR/TE = 3000ms/30ms, matrix = 64 × 64, voxel size = 3.3 mm × 3.3mm, flip angle = 80, slices = 6720, slice thickness = 3.3 mm.

2. The network-based statistics analysis

The network-based statistics (NBS) analysis was used to explore the significant changes in connectivity components between SCD and HC. First, independent statistical analysis was conducted on each connection, and the primary threshold ($p < 0.05$) was applied to identify suprathreshold connections and determine any connection components. Second, the nonparametric permutation approach was used to calculate the statistical significance of each observed component in the network (5000 permutations), and the empirical null distribution of the connected component size was derived. Finally, for a connected component of size N was found between the two

groups, and the proportion of 5000 permutations for which the maximal connected component was larger than N was used to determine the corrected *p* value.
