# Supplementary material for: Altered brain network dynamics and functional connectivity in subjective cognitive decline: an edge-centric network study
Source: Front Aging Neurosci. 2026 Jan 9;17:1596537. doi: 10.3389/fnagi.2025.1596537 (PMC12827695; doi:10.3389/fnagi.2025.1596537)
Supplement: Supplementary file 2 [file Table_1.docx]

| **Supplementary Table 1. Differences between SCD and HC network metrics in the edge-centric network based on Schaefer 100 atlas** | | | | | |
| --- | --- | --- | --- | --- | --- |
| measurements | SCD group (n=211) | HC group (n=210) | df | *p* value | Cohen's d (95% CI) |
| Peak amplitude | 90.75±2.89 | 91.45±3.21 | 419 | **0.019*** | **-0.227 (-0.419, -0.035)** |
| Trough-to-trough duration (TTD) | 5.94±4.92 | 4.92±4.08 | 419 | **0.022*** | **0.224(0.033, 0.415)** |
| ***High-amplitude frames network*** | | | | | |
| Normalized clustering coefficient (γ) | 0.83±0.17 | 0.87±0.17 | 419 | **0.043*** | **-0.198 (-0.390, -0.007)** |
| Nodal Betweenness in left precentral gyrus | 37.25±29.16 | 28.37±22.25 | 419 | **0.046*** | **0.342 (0.149, 0.534)** |
| Nodal Betweenness in right angular gyrus | 19.52±18.17 | 26.65±25.20 | 419 | **0.045*** | **-0.325 (-0.517, -0.132)** |
| Nodal Efficiency in right inferior frontal gyrus, triangular part | 0.25±0.05 | 0.26±0.04 | 419 | **0.049*** | **-0.1.98 (-0.3.90, -0.010)** |
| ***Low-amplitude frames network*** | | | | | |
| Normalized clustering coefficient (γ) | 0.71±0.09 | 0.73±0.11 | 419 | **0.017*** | **-0.233 (-0.424, -0.041)** |
| Small-worldness (σ) | 0.67±0.07 | 0.48±0.02 | 419 | **0.035*** | **-0.206(-0.397, -0.014)** |
| SCD, subjective cognitive decline; HC, healthy controls; df, degree of freedom; CI, confidence interval. | | | | | |
| The abnormal brain regions were determined if at least one of the three nodal centralities showed a significant between-group difference (*p* < 0.05, FDR corrected). | | | | | |
| *: *p* value < 0.05, FDR corrected. The values in bold indicate *p*-values and their confidence intervals for indicators with statistical differences. | | | | | |
